# A Review of the Tawny Crazy Ant, *Nylanderia fulva*, an Emergent Ant Invader in the Southern United States: Is Biological Control a Feasible Management Option?

**DOI:** 10.3390/insects7040077

**Published:** 2016-12-15

**Authors:** Zinan Wang, Lori Moshman, Emily C. Kraus, Blake E. Wilson, Namoona Acharya, Rodrigo Diaz

**Affiliations:** Department of Entomology, Louisiana State University Agricultural Center, Baton Rouge, LA 70803, USA; wangzinan2014@gmail.com (Z.W.); LMoshman@agcenter.lsu.edu (L.M.); ekraus@agcenter.lsu.edu (E.C.K.); bwils26@lsu.edu (B.E.W.); nachar1@lsu.edu (N.A.)

**Keywords:** classical biological control, invasive ant, *Solenopsis invicta*, *Pseudacteon* spp., *Myrmecomorba nylanderiae*

## Abstract

The tawny crazy ant, *Nylanderia fulva* (Mayr) (Hymenoptera: Formicidae), has invaded states of the U.S. including Texas, Louisiana, Mississippi, Alabama, Florida, and Georgia. Native to South America, *N. fulva* is considered a pest in the U.S. capable of annoying homeowners and farmers, as well as displacing native ant species. As it continues to expand its range, there is a growing need to develop novel management techniques to control the pest and prevent further spread. Current management efforts rely heavily on chemical control, but these methods have not been successful. A review of the biology, taxonomy, ecology, and distribution of *N. fulva*, including discussion of ecological and economic consequences of this invasive species, is presented. Options for future management are suggested focusing on biological control, including parasitoid flies in the genus *Pseudacteon*, the microsporidian parasite *Myrmecomorba nylanderiae*, and a novel polynucleotide virus as potential biological control agents. We suggest further investigation of natural enemies present in the adventive range, as well as foreign exploration undertaken in the native range including Paraguay, Brazil, and Argentina. We conclude that *N. fulva* may be a suitable candidate for biological control.

## 1. Introduction

Invasive species cause an estimated $120 billion in environmental damages annually in the United States alone [1]. Additionally, they are major factors in biodiversity loss and are the primary risk to 42% of the world’s threatened and endangered species [1]. The tawny crazy ant, *Nylanderia fulva* (Mayr) (Hymenoptera: Formicidae), is an invasive species which was first reported as a pest in the southern United States near Houston, Texas in 2002 [2]. This ant is now present in six southern states, and is already causing problems similar to those of the red imported fire ant, *Solenopsis invicta* (Buren). These problems include ecological damage such as reductions in diversity of native ants and other arthropods in invaded habitats [3], as well as potential economic damage for homeowners, public land owners, and farmers [4]. While ecological and economic impacts of *N. fulva* are comparable to that of *S. invicta*, the former does not sting and poses no medical threat to humans. The ant’s high likelihood of human-mediated dispersal [5] along with its association with hemipteran pests [6] give the pest potential to emerge as a threat to agricultural production. While there is not much information on the current status of *N. fulva* as an economic pest, we can draw similarities with other invasive ant species. In Texas alone *S. invicta* causes $671 million in annual damages and control costs in agriculture, households, golf courses, schools, and public places [4]. Public concern of *N. fulva* is on the rise as evidenced by an increase in news articles reporting local infestations. Increased public awareness of the impact of *N. fulva* may contribute to higher demand for effective control strategies.

Management of *N. fulva* relies on chemical and mechanical tools; however, these methods have not been effective at reducing populations [5]. This supports a need for alternative means of control. This review covers the biology, ecology, and impact of *N. fulva*, and discusses the potential for sustainable and effective management through biological control. We propose three natural enemies of *N. fulva* as potential biological control agents which should be investigated for their use in the United States. While these agents have biological control potential, there is still a need for domestic and foreign exploration for additional agents.

## 2. Biology

**Figure 1:**
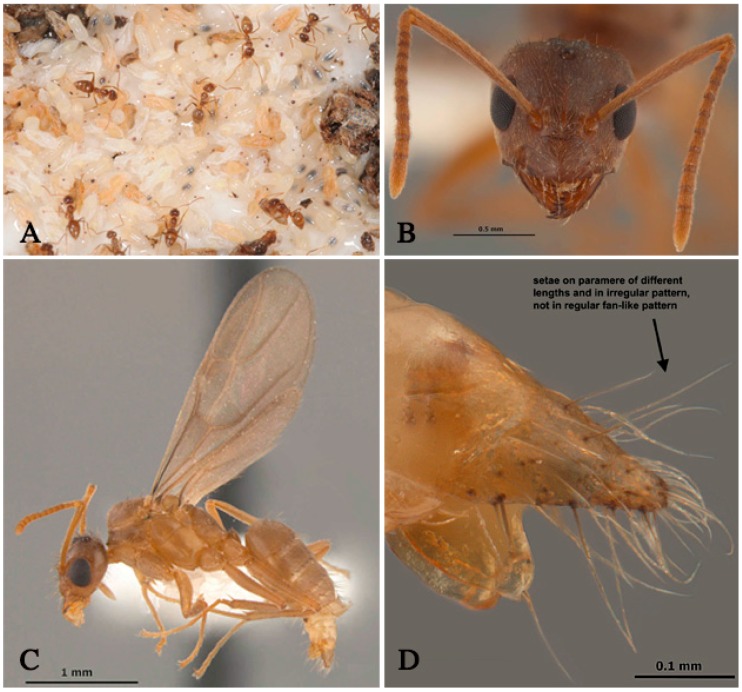
(**A**) *Nylanderia fulva* workers with brood; (**B**) Full face view of *N. fulva* female; (**C**) Lateral view of *N. fulva* male; (**D**) Lateral view of *N. fulva* male paramere. Picture A was taken by Blake Layton (Mississippi State University Extension), and (**B**–**D**) were taken by Joe A. MacGown (Mississippi Entomological Museum).

Queens of *N. fulva* lay single white ovoid-shaped eggs (0.4 mm in length) which are then attached to the egg mass (17–25 eggs) with saliva by workers [7,8]. The mean duration of the egg stage is 16.2 days and is followed by the emergence of hymenopteriform larvae which pass through three instars for workers and four instars for males, lasting 11 days [7]. Following pupation, workers carry pupae to dryer portions of the nest where they are piled into mounds. Exarate pupae (2.6 mm) are initially white before changing to light brown (Figure 1A), and adult emergence occurs after 12 days [7]. Adults are workers, males, or queens. While the occurrence of alate males and females has been documented [2,5], nuptial flight activity had not been recorded until recently. New findings have confirmed that alate males actively fly throughout the year with a peak in abundance during the summer. Alate females are produced once a year in late summer, but flight activity of females has not been confirmed. It is likely that queens attract flying males via pheromonal cues to facilitate colony reproduction through budding [9].

## 3. Taxonomy

*Nylanderia* belongs to the Lasiini tribe of the subfamily Formicinae [10]. Workers of *Nylanderia* spp. are characterized by the presence of six (rarely seven) mandibular teeth, six-segmented maxillary palps, and four-segmented labial palps [11] (Figure 1B). The dorsum of the mesosoma as well as the head, scapes, and legs are setose, whereas the propodeum commonly lacks setae [11]. The eyes are well developed and spaced laterally on the head (Figure 1B,C). Like other members of the *Prenolepis* genus-group, *Nylanderia* spp. have triangular-shaped mandibles and 12-segmented antennae [12] (Figure 1B). *Nylanderia fulva* workers are light brown in color and monomorphic, with an average body size of 2 mm [2,5,11,13] (Figure 1A). Males range from 2.4–2.7 mm in length (Figure 1C) while queens are approximately 4.0 mm or longer [14].

The genus *Nylanderia* has undergone several revisions. *Nylanderia* was formerly regarded as a subgenus of *Paratrechina* and was elevated to its own genus on several different occasions [15]. The most recent revision by LaPolla et al. (2010; 2011) [11,12] found *Paratrechina* to be polyphyletic and it was divided into three monophyletic genera: *Paratrechina*, *Paraparatrechina*, and *Nylanderia*, with most of the members of the original *Paratrechina* placed in *Nylanderia*. *Nylanderia* forms a monophyletic clade with *Paratrechina* and two other genera, *Pseudolasius* and *Euprenolepis*. The genus *Nylanderia* has a wide tropical and subtropical distribution [11]. More recent studies of the genus have shown *Nylanderia* is also present in the Nearctic region. This includes North America as far south as northern Mexico and as far north as Greenland. There are 20 species of *Nylanderia* present in the United States (Table 1). These include 14 native species which should be considered as potential non-target species when implementing a biological control agent [16]. Additionally, there are six species which have been introduced to the United States, but only *N. fulva* is considered a pest [16].

*Nylanderia fulva* in the U.S. was originally identified incorrectly as *Paratrechina* sp. nr. *pubens*, and later revised to *N. fulva* [11,13]. It was also discovered that the ant species identified as *N.* cf. *pubens* in Florida was, in fact, *N. fulva* [2,17]. *Nylanderia pubens* and *N. fulva* are very closely related, together forming a monophyletic group [13]. Due to a lack of distinctive characters in the workers, these two species are often confused or misidentified [13]. Males of *N. fulva* are distinguished from *N. pubens* by their triangular and weakly sclerotized parameres with few erect setae [13] (Figure 1D). The difficulty in making correct taxonomic identifications of *Nylanderia* species has greatly hindered the research of *Nylanderia* [11]. There are several different common names in reference to *N. fulva*, including Rasberry crazy ant, hairy crazy ant, and Caribbean crazy ant. However, the official common name recognized by the Entomological Society of America is tawny crazy ant [18].

## 4. Distribution and Spread

*Nylanderia fulva* is native to South America, specifically southern Brazil and northern Argentina along the border of Uruguay and Paraguay [13,19]. Along with these countries, this ant has become established in Anguilla, Bermuda, Colombia, Cuba, Guadeloupe, Martinique, Mexico, Panama, Puerto Rico, St. Vincent and the Grenadines (Lesser Antilles), and the U.S. Virgin Islands [13,15,20,21]. Based on the species occurrences in the invaded and native range, Kumar et al. (2015) predicted the potential distribution of *N. fulva* to cover most of Central and South America; central Africa, and South Asia [19].

**Figure 2:**
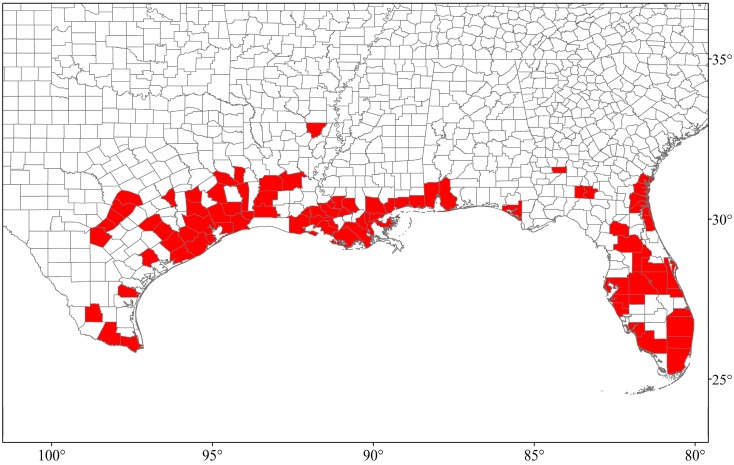
Current known distribution of *N. fulva* in counties of Texas, Louisiana, Mississippi, Alabama, Florida, and Georgia as of September, 2016 [13,14,22].

Once established in an area, spread of *N. fulva* occurs when new colonies form from colony fission or from movement of transitory nests. Invasive populations in Texas spread at a rate of 20–30 m per month during 2002–2006 [2], while rates of range expansion in Colombia of approximately 100 m per month have been reported [8]. Range expansion at much greater rates can result from human-aided dispersal. Subsequent introductions have allowed *N. fulva* to become established in six states: Texas, Louisiana, Mississippi, Alabama, Florida, and Georgia (Figure 2) [13,14,22]. Potential distribution for *N. fulva* in the U.S. has not yet been determined. However, *N. fulva* has a greater lower critical thermal limit (≈7 °C) than *S. invicta* (≈4 °C) [23]. Thus, *N. fulva* is expected to be limited to areas further south than the distribution of *S. invicta*, which reaches as far north as southern Tennessee, Arkansas, and Oklahoma [24].

## 5. Ecology

*Nylanderia fulva* larvae are raised in either transitory or permanent nests typically occurring in humid soil with tunnels or crevices created by other arthropods [8]. Transitory nests containing only workers and larvae on the soil surface are most common during rainy conditions and are moved almost daily. Permanent nests are found in well drained areas and may cover an area as large as one square meter. These nests contain all castes and life stages, and immatures may be kept as deep as 40 cm below the soil surface [8]. Nests are most often polygynous with typically 0–5 queens, and reproduction capacity increases with increasing number of queens [5,7]. Transitory nests are responsible for range expansion and are more prevalent than permanent nests in newly invaded areas [8]. *Nylanderia fulva* forms super-colonies and no aggression is observed between nests [3,25].

The diet of *N. fulva* is similar to other omnivorous ants and consists of protein from predation and scavenging of arthropods and higher animals along with carbohydrates from liquid exudates from plants or hemipteran insects [8]. Predation of a wide range of arthropods has been observed, but most of the diet is composed of Lepidoptera, Coleoptera, Isoptera, and Hymenoptera including many other ant species [8]. Higher animals which may be preyed upon are birds (including chickens), small mammals, snakes, and lizards [8]. While this ant can be beneficial in some agricultural systems by consuming larvae of coleopteran and lepidopteran pest species, the tendency of *N. fulva* to form symbiotic relationships with plant-feeding hemipterans can enhance pest problems [6]. Associations with hemipterans involve protection and transportation of hemipteran colonies while obtaining carbohydrates from honeydew produced by the plant-feeding insect. *Nylanderia fulva* is known to protect hemipterans by preying on their natural enemies and by constructing shelters from soil particles over active hemipteran colonies [6,8]. These symbiotic relationships are formed with species from seven families of Hemiptera: Aleyrodidae, Aphididae, Coccidae, Kermesidae, Pseudococcidae, Psyllidae, and Tingidae [6].

## 6. Ecological Impact

Ecological impacts of invasion by *N. fulva* include reductions in arthropod diversity, particularly on native ant assemblages. Once established in a new area, supercolonies of *N. fulva* can become extremely dominant, and some have been recorded in Texas reaching biomasses of more than two orders of magnitude greater than all other ant populations combined [3]. Competition with other ant species can occur indirectly through consumption of shared resources or through interference involving direct clashes with, and predation of, colonies of other ant species. *Nylanderia fulva* outcompetes other invasive ants including *Atta* spp. and *S. invicta* [3,8,25,26,27], and the impact of *N. fulva* is greater on smaller ant species than larger ones [28,29]. In Colombia, 9 out of 14 native species were displaced following establishment of *N. fulva* [29]. While *S. invicta* is also an aggressive invasive ant which has become the dominant ant species across the southeastern United States, co-evolution of these two species in their native range in Argentina appears to have given *N. fulva* the competitive advantage [3,26]. Populations of *S. invicta* frequently decline or are eliminated from areas following establishment of *N. fulva*. In locations where both species are present in similar numbers, *N. fulva* captures >90% of food resources [3,25,26]. While both species use venom as their primary weapon in these clashes, *N. fulva* has the ability to detoxify *S. invicta* venom, an important factor contributing to the competitive displacement of both native and invasive ant species [25,26].

In addition to disruption of arthropod communities, introduced populations of *N. fulva* in Colombia have caused desiccation of rangeland grasses through their associations with phytophagous hemipteran species [6,8]. This invasive ant undoubtedly impacts ecosystems in its adventive range and has the potential to cause cascading ecological impacts [3,29]. The highly aggressive and competitive nature of *N. fulva* may lead to even greater impacts than have been observed with other damaging invasive ants such as *S. invicta*.

## 7. Economic Impacts and Management

The tendency for large population densities of *N. fulva* to occur in a variety of areas including businesses and homes, makes this species an urban pest, and many people are seeking solutions to remove the ants from their property [2]. Besides their sheer numbers, *N. fulva* colonies have been reported to affect homeowners because their foraging and nesting behavior can cause short circuits in electrical equipment [2]. This species affects public areas such as parks and schools in similar ways [2]. Although the extent and diversity of damage caused by *N. fulva* has not been thoroughly assessed, it will likely be comparable to *S. invicta* which is known to cause damage to household structures, golf courses, schools, and infrastructure including irrigation systems and lighting fixtures [4]. Models indicate the total economic impact of *S. invicta* is greater than $6 billion for the United States and Puerto Rico [30]. Additionally, the association of *N. fulva* with a wide range of hemipteran pests may disrupt natural biological control and result in revenue losses from increased crop damage [6]. For example, increased economic losses from hemipteran pests in coconuts in the Caribbean [20] and coffee in South America have been attributed to associations with *N. fulva* [8]. The economic impacts of *N. fulva* in other agricultural habitats including row crops, orchards, and livestock grazing lands have not yet been quantified. Other areas in need of assessment are damage and control costs incurred in households, golf courses, schools, and other urban environments. Models which were developed to assess economic impacts of *S. invicta* can likely be adapted to quantify the damage associated with invasive populations of *N. fulva*.

The control tactics used against other invasive ants are not effective against *N. fulva* [5], and large scale suppression of populations in the United States has not been documented. Current control for *N. fulva* involves a combination of chemical and mechanical tactics. Mechanical control relies on removal of potential food sources and harborage (leaf litter and other yard debris) along with securing potential entry points into the home [31]. There are multiple pesticides that can be used as part of a management program including chlorfenapyr, fipronil, dinotefuran, and bifenthrin [31]. After chemical applications, large amounts of ant carcasses must be removed, and broad spectrum insecticides such as these are not suitable for many natural environments due to the risk of non-target effects on bees and other organisms [32]. Insecticides formulated as baits have been effective in controlling *S. invicta* with reduced ecological consequences relative to insecticides with contact toxicity [4]. However, many insecticidal baits which are effective against *S. invicta* are not attractive to *N. fulva* [2]. An insecticidal bait formulated for carpenter ants containing abamectin was shown to reduce *N. fulva* populations by >50%, however, this level of control was not considered acceptable and population suppression was not sustained [5]. Further, the economics of chemical control have not been examined, and high costs of insecticidal treatments are a major contributor to the economic impact of invasive ants [4]. Effective management strategies must be developed to sustainably suppress populations of *N. fulva* with less risk of ecological and environmental impacts. Biological control has potential to provide sustainable long-term suppression of *N. fulva* throughout the southeastern United States.

## 8. Biological Control Candidates

We have identified three potential natural enemies reported to attack *N. fulva*. A phorid fly, *Pseudacteon convexicauda* Borgmeier (Diptera: Phoridae), has been reported to parasitize *N. fulva* in Brazil and Argentina [33,34,35]. Phorid flies are effective natural enemies against ants and many are specific to a particular ant genus. For instance, *Pseudacteon curvatus* (Borgmeier) specifically parasitizes imported fire ants, *Solenopsis* spp. [36]. This species has now been released in the southeastern United States as a classical biological control agent for *S. invicta* [37]. *Pseudacteon* spp. which attack *N. fulva* in its native as well as adventive ranges should be explored as potential biological control candidates.

A newly described microsporidian, *Myrmecomorba nylanderiae* gen. et sp. nov., was found infecting *N. fulva* populations in Texas, Florida, and St. Croix in the U.S. Virgin Islands [38]. It produces three types of spores which infect the fat bodies of all *N. fulva* life stages [38]. High infection rates (≈70%) observed in all *N. fulva* populations examined suggest the microsporidium is readily transmitted among individuals in the polydomous supercolonies [38]. Although mortality from *M. nylanderiae* has not been studied at a colony level, reduced melanization and expanded intersegmental membranes were observed in infected *N. fulva* workers [38]. A similar microsporidium, *Kneallhazia solenopsae*, has been shown to cause high mortality in *S. invicta* colonies [39]. Moreover, an 85%–100% reduction in brood production and higher queen mortality resulted from infection of *S. invicta* colonies by *K. solenopsae* under laboratory conditions [40]. These microsporidians have the potential to be developed as biopesticides [35]. However, more research is needed to determine feasibility and efficacy of *M. nylanderiae* as a control agent of *N. fulva*, as well as non-target impacts.

A novel virus capable of infecting *N. fulva* has been isolated, and its polynucleotide and amino acid sequences have been identified [41]. The virus is present in ant populations in Argentina, but absent from United States populations, and is thought to be relatively host specific and have the potential to be developed for use as a biopesticide [41]. Additional entomopathogens and viruses infecting *N. fulva* in South America may also have value as biological agents or biopesticides.

Explorations for natural enemies which coevolved with *N. fulva* in its native range may provide promising candidates for classical biological control. Several locations in South America have a similar climate to the southeastern United States including northern Argentina, Paraguay, and southern Brazil [19]. These regions would offer the best likelihood for finding effective biological control agents of *N. fulva* in its native range. To facilitate the exploration of natural enemies, we suggest conducting genetic studies to determine the origin of the United States populations. In addition, natural enemies already present in the United States may contribute to managing this invasive species.

Direct mortality or stress from biological control agents could reduce the fitness and competitive ability of *N. fulva*, ultimately reducing the ecological and economic impacts of this invasive pest. The use of biological control can also reduce the reliance on chemical agents and decrease non-target effects [42]. However, biological control programs, particularly those involving the introduction of exotic species, are not without risk, and risk assessment and management is critical before any action is taken [42]. After searching for natural enemies of *N. fulva*, candidate biological control agents should be selected carefully with consideration of the potential non-target risk. Due to the number of native species in the genus *Nylanderia* in North America, the host specificity of agents must be carefully studied. Protocol for assessing host specificity is reviewed by Babendreier et al. [43] and typically involves choice and no-choice experiments with appropriate potential host species. Host range data have been widely used to analyze the direct risks of the introductions in classical biological control programs [42].

## 9. Conclusions and Future Research

Native to Brazil and Argentina, *N. fulva* has become an invasive ant in many regions of North, Central, and South America, as well as the Caribbean. Since it was first detected in the United States in 2002, the ant has spread to six southeastern states where it is responsible for considerable economic and ecological impacts in agricultural and urban habitats. The widespread damage resulting from the invasion of the southeastern United States by *S. invicta* demonstrates the potential of exotic ant species to have dramatic impacts in their adventive range. The highly competitive nature of *N. fulva* allows it to suppress other native and introduced ant species, including *S. invicta*, and to reach damaging population densities. The ecological impacts of *N. fulva* in its adventive range will require detailed investigation. Additionally, extensive quantification of economic impacts and control costs of *N. fulva* in the United States is needed. These should include examination of potential increases in crop losses from hemipteran pests resulting from associations with *N. fulva*. Although chemical and mechanical control methods have been able to reduce *N. fulva* populations in some instances, sustainable long-term management strategies are needed to mitigate the pest’s impact. Biological control has the potential to provide area-wide suppression and help restore invaded ecosystems. Potential natural enemies which may be used as biological control agents include the phorid fly *Pseudacteon convexicauda*, the microsporidian parasite *Myrmecomorba nylanderiae*, and a novel polyhedral virus. The native range of *N. fulva* in Brazil and Argentina should be explored for host specific natural enemies including *Pseudacteon* spp. and other arthropods. Additionally, the potential to develop *M. nylanderiae* and novel viruses as biopesticides for control of *N. fulva* should be further examined. We believe that *N. fulva* may be a suitable candidate for biological control, but more research and exploration will be needed before potential biological control programs can be developed. Managing *N. fulva* in managed and natural ecosystems will require the integration of several approaches including the prevention of arrival to new regions, the use of registered chemicals and mechanical controls, and the delivery of host specific natural enemies.

## Figures and Tables

**Table 1:** Distribution of species in the genus *Nylanderia* and their classification as native or invasive in the United States [16].

Species	Native/Invasive	Range	Overlap with *N. fulva*
*N. arenivaga*	Native	Eastern and central U.S.	Yes
*N. austroccidua*	Native	Southwest U.S., Mexico to Costa Rica	Yes
*N. bruesii*	Native	Southwest U.S., northwestern Mexico	Yes
*N. concinna*	Native	Eastern U.S.	Yes
*N. faisonensis*	Native	Eastern U.S.	Yes
*N. hystrix*	Native	Southwestern U.S.	Yes
*N. magnella*	Native	Southwestern U.S.	Yes
*N. parvula*	Native	Eastern U.S.	Yes
*N. phantasma*	Native	Southeastern U.S.	Yes
*N. querna*	Native	Central U.S.	Yes
*N. terricola*	Native	U.S. and northern Mexico	Yes
*N. trageri*	Native	Central U.S.	Yes
*N. vividula*	Native	U.S. and northern Mexico	Yes
*N. wojciki*	Native	Southeastern U.S.	Yes
*N. bourbonica*	Introduced	Florida	Yes
*N. flavipes*	Introduced	Northeastern U.S.	No
*N. guatemalensis*	Introduced	Florida	Yes
*N. pubens*	Introduced	Florida	Yes
*N. steinheili*	Introduced	Florida	Yes

## References

[1] Pimentel D., Zuniga R., Morrison D. (2005). Update on the environmental and economic costs associated with alien-invasive species in the United States. Ecol. Econ..

[2] Meyers J.M. (2008). Identification, Distribution and Control of an Invasive Pest Ant, *Paratrechina* sp. (Hymenoptera: Formicidae), in Texas. Ph.D. Thesis.

[3] LeBrun E.G., Abbott J., Gilbert L.E. (2013). Imported crazy ant displaces imported fire ant, reduces and homogenizes grassland ant and arthropod assemblages. Biol. Invasions.

[4] Lard C., Willis D.B., Salin V., Robison S. (2002). Economic assessments of red imported fire ant on Texas’ urban and agricultural sectors. Southwest Entomol..

[5] McDonald D.L. (2012). Investigation of an Invasive Ant Species: *Nylanderia fulva* Colony Extraction, Management, Diet Preference, Fecundity, and Mechanical Vector Potential. Ph.D. Thesis.

[6] Sharma S., Oi D.H., Buss E.A. (2013). Honeydew-producing hemipterans in Florida associated with *Nylanderia fulva* (Hymenoptera: Formicidae), an invasive crazy ant. Fla. Entomol..

[7] Arcila A.M., Gómez L.A., Ulloa-Chacón P. (2002). Immature development and colony growth of crazy ant *Paratrechina fulva* under laboratory conditions (Hymenoptera: Formicidae). Sociobiology.

[8] Zenner-Polanía I., Meer R.K.V., Jaffe K., Cedeno A. (1990). Biological aspects of the “hormiga loca”, *Paratrechina* (*Nylanderia*) *fulva* (Mayr), in Colombia. Applied Myrmecology: A World Perspective.

[9] McDonald D.L. (2016). Personal communication.

[10] Brady S.G., Schultz T.R., Fisher B.L., Ward P.S. (2006). Evaluating alternative hypothese for the early evolution and diversification of ants. Proc. Natl. Acad. Sci. USA.

[11] LaPolla J., Brady S.G., Shattuck S.O. (2011). Monograph of *Nylanderia* (Hymenoptera: Formicidae) of the world: An introduction to the systematics and biology of the genus. Zootaxa.

[12] LaPolla J., Brady S.G., Shattuck S.O. (2010). Phylogeny and taxonomy of the *Prenolepis* genus-group of ants (Hymenoptera: Formicidae). Syst. Entomol..

[13] Gotzek D., Brady S.G., Kallal R.J., LaPolla J.S. (2012). The importance of using multiple approaches for identifying emerging invasive species: The case of the Rasberry crazy ant in the United States. PLoS ONE.

[14] MacGown J., Layton B. (2010). The invasive Rasberry crazy ant, *Nylanderia* sp. Near *pubens* (Hymenoptera: Formicidae), reported from Mississippi. Midsouth Entomol..

[15] Trager J.C. (1984). A revision of the genus *Paratrechina* of the continental United States. Sociobiology.

[16] Kallal R.J., Lapolla J.S. (2012). Monograph of *Nylanderia* (Hymenoptera: Formicidae) of the world, part II: *Nylanderia* in the Nearctic. Zootaxa.

[17] Zhao L., Chen J., Jones W.A., Oi D.H., Drees B.M. (2012). Molecular comparisons suggest Caribbean crazy ant from Florida and Rasberry crazy ant from Texas (Hymenoptera: Formicidae: *Nylanderia*) are the same species. Environ. Entomol..

[18] Entomological Society of America (2016). Common Names of Insects Database. http://www.entsoc.org/common-names.

[19] Kumar S., LeBrun E.G., Stohlgren T.J., Stabach J.A., McDonald D.L., Oi D.H., LaPolla J.S. (2015). Evidence of niche shift and global invasion potential of the tawny crazy ant, *Nylanderia fulva*. Ecol. Evol..

[20] Wetterer J.K., Keularts J.L. (2008). Population explosion of the hairy crazy ant, *Paratrechina pubens* (Hymenoptera: Formicidae), on St. Croix, US virgin islands. Fla. Entomol..

[21] Hill S.K., Baldwin R.W., Pereira R.M., Koehler P.G. Tawny Crazy Ant. http://edis.ifas.ufl.edu/pdffiles/IN/IN107600.pdf.

[22] Graham L.C., Buss L., Henderson G., Suiter D.R., Puckett R.T., Layton B. (2016). Personal communication.

[23] Bentley M., Hahn D., Oi F. (2016). The thermal breadth of *Nylanderia fulva* (Hymenoptera: Formicidae) is narrower than that of *Solenopsis invicta* at three thermal ramping rates: 1.0, 0.12, and 0.06 °C·min^−1^. Environ. Entomol..

[24] USDA (2016). Species Profiles: Red Imported Fire Ant. https://www.invasivespeciesinfo.gov/animals/rifa.shtml.

[25] LeBrun E.G., Jones N.T., Gilbert L.E. (2014). Chemical warfare among invaders: A detoxification interaction facilitates an ant invasion. Science.

[26] Horn K. (2009). Examining Competitive Interaction between Rasberry Crazy Ants (*Paratrechina* sp. Nr. *Pubens*) and Red Imported Fire Ants (*Solenopsis invicta*) Using Laboratory and Field Studies. Master’s Thesis.

[27] Chen J., Rashid T., Feng G., Zhao L., Oi D. (2013). Defensive chemicals of tawny crazy ants, *Nylanderia fulva* (Hymenoptera: Formicidae) and their toxicity to red imported fire ants, *Solenopsis invicta* (Hymenoptera: Formicidae). Toxicon.

[28] Horn K.C., Eubanks M.D., Siemann E. (2013). The effect of diet and opponent size on aggressive interactions involving Caribbean crazy ants (*Nylanderia fulva*). PLoS ONE.

[29] Zenner-Polania I., Williams D.F. (1994). Impact of *Paratrechina fulva* on other ant species. Exotic Ants: Biology, Impact and Control of Introduced Species.

[30] Lard C., Schmidt J., Morris B., Estes L., Ryan C., Berquist D. (2006). An Economic Impact of Imported Fire Ants in the United States of America.

[31] Oi F., Calibeo D., Paige J., Bentley M. (2016). Integrated Pest Management (IPM) of the Tawny Crazy Ant, Nylanderia fulva (Mayr).

[32] Sanchez-Bayo F., Goka K. (2014). Pesticide residues and bees–a risk assessment. PLoS ONE.

[33] Porter S.D., Pasquero M.A. (2002). Illustrated key to *Pseudacteon* decapitating flies (Diptera: Phoridae) that attack *Solenopsis saevissima* complex fire ants in South America. Fla. Entomol..

[34] Pasquero M.A. (2016). Personal communication.

[35] Brown B.V., Schneider S.A., LaPolla J.S. (2011). A new north American species of *Pseudacteon* (Diptera: Phoridae), parasitic on *Nylanderia arenivaga* (Hymenoptera: Formicidae). Ann. Entomol. Soc. Am..

[36] Porter S.D. (2000). Host specificity and risk assessment of releasing the decapitating fly *Pseudacteon curvatus* as a classical biocontrol agent for imported fire ants. Biol. Control.

[37] Graham L.C., Porter S.D., Pereira R.M., Dorough H.D., Kelley A.T. (2003). Field releases of the decapitating fly *Pseudacteon curvatus* (Diptera: Phoridae) for control of imported fire ants (Hymenoptera: Formicidae) in Alabama, Florida, and Tennessee. Fla. Entomol..

[38] Plowes R.M., Becnel J.J., LeBrun E.G., Oi D.H., Valles S.M., Jones N.T., Gilbert L.E. (2015). *Myrmecomorba nylanderiae* gen. et sp. nov., a microsporidian parasite of the tawny crazy ant *Nylanderia fulva*. J. Invertebr. Pathol..

[39] Knell J., Allen G., Hazard E. (1977). Light and electron microscope study of *Thelohania solenopsae* n. Sp. (Microsporida: Protozoa) in the red imported fire ant, *Solenopsis invicta*. J. Invertebr. Pathol..

[40] Oi D.H., Williams D.F. (2002). Impact of *Thelohania solenopsae* (microsporidia: Thelohaniidae) on polygyne colonies of red imported fire ants (Hymenoptera: Formicidae). J. Econ. Entomol..

[41] Valles S.M., Oi D.H., Becnel J.J., Wetterer J.K., LaPolla J.S., Firth A.E. (2016). Isolation and characterization of *Nylanderia fulva* virus 1, a positive-sense, single-stranded RNA virus infecting the tawny crazy ant, *Nylanderia fulva*. Virol. J..

[42] Van Lenteren J., Bale J., Bigler F., Hokkanen H., Loomans A. (2006). Assessing risks of releasing exotic biological control agents of arthropod pests. Annu. Rev. Entomol..

[43] Babendreier D., Bigler F., Kuhlmann U. (2005). Methods used to assess non-target effects of invertebrate biological control agents of arthropod pests. BioControl.

